# Diffuse Bone Marrow Metastasis as the Initial Presentation of an Occult Breast Cancer

**DOI:** 10.1155/2018/2946409

**Published:** 2018-07-22

**Authors:** Frank S. Fan, Chung-Fan Yang, Yi-Fen Wang

**Affiliations:** ^1^Section of Hematology and Oncology, Department of Medicine, Changhua Hospital, Ministry of Health and Welfare, Chang-Hua County, Taiwan; ^2^Department of Pathology, Changhua Hospital, Ministry of Health and Welfare, Chang-Hua County, Taiwan; ^3^Department of Radiology, Changhua Hospital, Ministry of Health and Welfare, Chang-Hua County, Taiwan

## Abstract

**Introduction:**

Breast cancer is one of the malignancies which tend to involve the bone marrow, but initial presentation with diffuse bone marrow metastasis from an occult breast cancer is very rare. Prognosis is generally very poor for marrow metastasis from solid tumors except that breast cancer is a treatable disease even in such a dismal condition.

**Case:**

A 64-year-old woman's headache was found to result from diffuse adenocarcinoma metastasis in the bone marrow from an unknown primary site. Intensive immunohistochemistry study of bone marrow biopsy specimen confirmed the disease nature to be an estrogen receptor-positive/human epidermal growth factor receptor 2-negative breast cancer. Mammography and magnetic resonance imaging of breasts revealed a suspicious primary lesion in the right breast. Treatment with tamoxifen alone achieved a sustained response.

**Discussion:**

Mucin 1 (MUC1), also known as cancer antigen 15-3 (CA 15-3), facilitates motility and metastatic potential of breast cancer cells. Interleukin-1*β* (IL-1*β*) drives breast cancer cell growth and colonization in bone marrow adipose tissue niche. Receptor activator of nuclear factor kappa-B (RANK) and its ligand (RANKL) activate osteoclasts to make a favorable bone marrow microenvironment for tumor cells. Agents against MUC1, IL-1*β*, and RANKL might be of therapeutic effect for patients like ours.

## 1. Introduction

Bone marrow metastasis from nonhematological tumors is a well-known event. The most common origins identified are lung, breast, stomach, and prostate [1, 2]. But the primary sites of a large portion frequently still remain unknown with generally unfavorable prognosis [3, 4]. Nevertheless, symptomatic bone marrow metastasis from breast cancer seems to have a relatively longer median survival, probably due to its treatable characteristics [5]. We like to present a case of marrow carcinomatosis which was proven to originate from a hard-to-identify breast cancer by immunohistochemical staining and responded dramatically to tamoxifen alone. The possible underlying mechanisms of stealthy bone marrow metastasis from an occult breast cancer and recent progress in management of such a disastrous status will also be discussed.

## 2. Case Presentation

A 64-year-old woman was brought to the emergency unit with chief complaints of headache and fever for one week in September 2017. She felt pain over her whole calvarium without a specific trigger point. Her body temperature was 38.7 degrees Celsius. There was no nausea, vomiting, blurred vision, or nuchal stiffness. Cranial and peripheral neurologic function did not have any impairment. Superficial lymphadenopathy, breast nodules, and abdominal tumor mass were not detected on palpation. A Babinski sign was absent. She denied drug or alcohol abuse.

Laboratory examination disclosed a normocytic anemia with hemoglobin level of 7 g/dl, mean corpuscular volume of 89.3 fl, platelet count of 325000/*μ*l, and white blood cell count of 12000/*μ*l comprising neutrophils 61.7%, lymphocytes 26.7%, and monocytes 10.8%. A leucoerythroblastic picture was not present. Blood chemistry tests revealed abnormally elevated serum alkaline phosphatase of 158 iu/l (normal 32~91) and lactate dehydrogenase of 292 iu/l (normal 98~192). There was neither microhematuria nor stool occult blood. Both activated partial thromboplastin time and prothrombin time were normal in value but the D-dimer was extremely high: 6570 ng/ml (normal 0~500).

**Figure 1:**
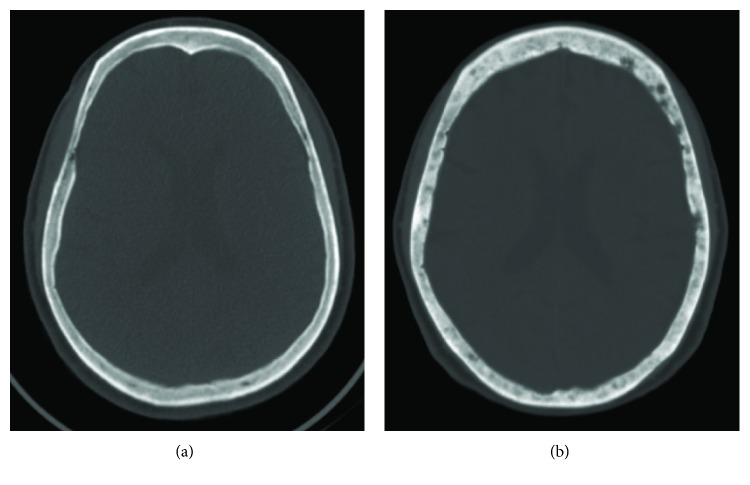
Axial head CT images on bone window. (a) The skull was intact on June 30, 2011. (b) Many osteolytic lesions were detected on September 13, 2017.

The chest X-ray routine film showed no active lung lesions. A computerized tomography (CT) scan of head was ordered to rule out intracranial abscess or other central nervous system problems. The brain turned out to be intact but, unexpectedly, multiple osteolytic lesions were detected in the skull, extraordinarily obvious upon comparison with previous films taken six years earlier for other reasons (Figure 1). She was then admitted to the ward under a suspicious impression of multiple myeloma.

**Figure 2:**
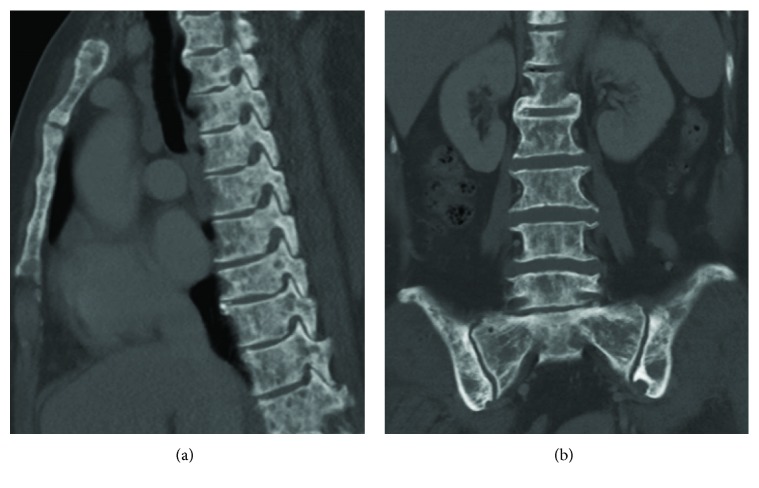
Chest and abdomen CT images on bone window, September 14, 2017. Multiple osteosclerotic and osteolytic lesions involved the sternum, vertebra, sacrum, and bilateral iliac bones.

Subsequent immunofixation electrophoresis analysis of serum, however, did not show any evidence of monoclonal gammopathy, and the levels of serum immunoglobulin G, A, and M were all within normal ranges. In contrast, serum tumor markers carcinoembryonic antigen (CEA) and cancer antigen 15-3 (CA 15-3) were meaningfully increased in concentration: CEA 8.1 ng/ml (normal 0~5) and CA 15-3 163.2 iu/ml (normal 0~31.3), respectively. Levels of cancer antigen 19-9 (CA 19-9) and cancer antigen 125 (CA-125) were within normal limits. Afterwards, a CT scan of chest and abdomen revealed osteosclerotic and osteolytic lesions similar to that of skull involving almost the whole skeleton (Figure 2) but not a single clear-cut primary tumor site could be located.

**Figure 3:**
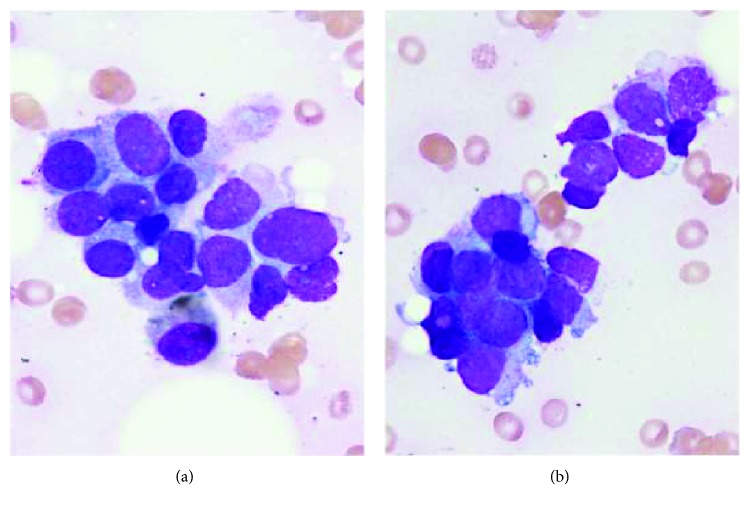
Adenocarcinoma nests in bone marrow aspiration smears (Wright-Giemsa stain, 1000x).

Bone marrow aspiration and biopsy were performed on the right side posterior superior iliac crest. The aspiration smear demonstrated very scanty hematopoietic precursors scattered among crowded groups of oval neoplastic cells with large hyperchromatic nuclei, coarse chromatin, modest granular cytoplasm, and occasionally small nucleoli (Figure 3). Frequent rosette-like openings were seen in the tumor groups. A metastatic adenocarcinoma was considered as the most likely diagnosis.

**Figure 4:**
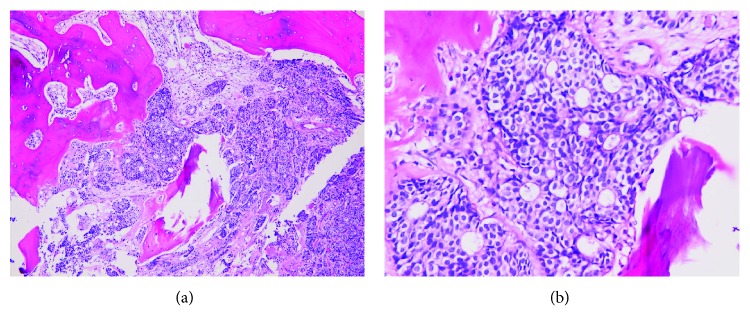
Metastatic adenocarcinoma in bone marrow (Hematoxylin and eosin stain, (a) 100x; (b) 400x).

**Figure 5:**
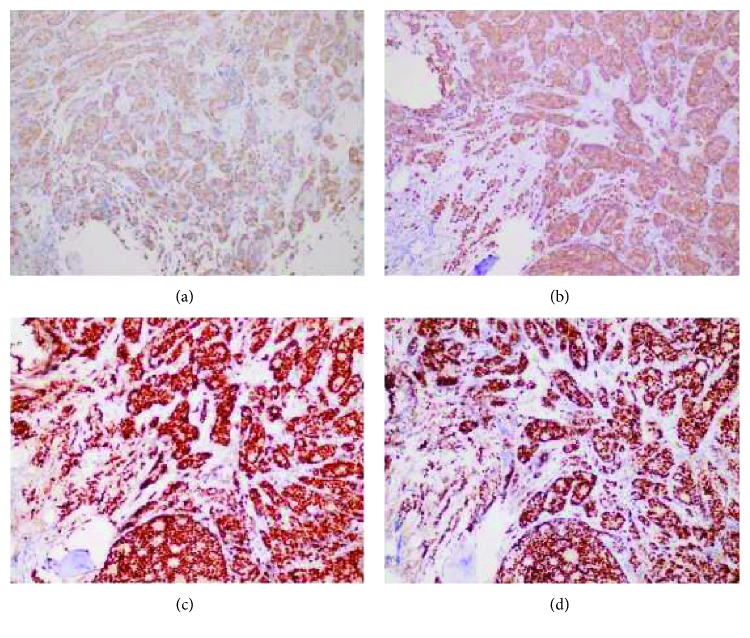
Immunohistochemical stain of adenocarcinoma in bone marrow. (a) CK7, (b) CK8, (c) ER, and (d) PR. All were positive.

**Figure 6:**
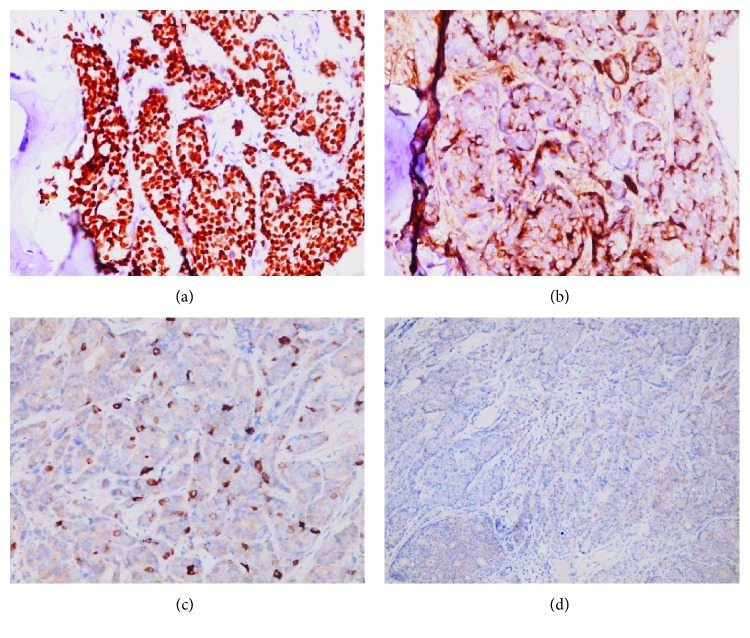
Immunohistochemical stain of adenocarcinoma in bone marrow. Positive for (a) GATA-3, (b) GCDFP-15 (patchy staining pattern), and (c) mammaglobin (scattered staining pattern). Negative for (d) HER2.

Pathologic study of the biopsy specimen after decalcification displayed a picture of metastatic adenocarcinoma composed of round to oval tumor cells arranged in ovoid clusters, small rounded nests, cribriform nests, and focal microacinar pattern stuffed in bone marrow cavity (Figure 4). The immunohistochemical stains gave positive results for cytokeratin 7 (CK7), cytokeratin 8 (CK8), estrogen receptor (ER) (strong, 99%), progesterone receptor (PR) (strong, 99%) (Figure 5), Smad4 (DPC4), GATA binding protein 3 (GATA-3), gross cystic disease fluid protein 15 (GCDFP-15), and mammaglobin (Figure 6). While human epidermal growth factor receptor 2 (HER2/neu), cytokeratin 20 (CK20), thyroid transcription factor-1 (TTF-1), paired box gene 8 (PAX8), and synaptophysin were negative. The positive rate of Ki-67 in tumor cells was 20%. Diffuse bone marrow metastasis from breast carcinoma (invasive ductal carcinoma, not otherwise specified) thus should be considered firstly as the diagnosis based on aforementioned pathological findings and differential diagnosis markers recommended in the literature [6, 7].

**Figure 7:**
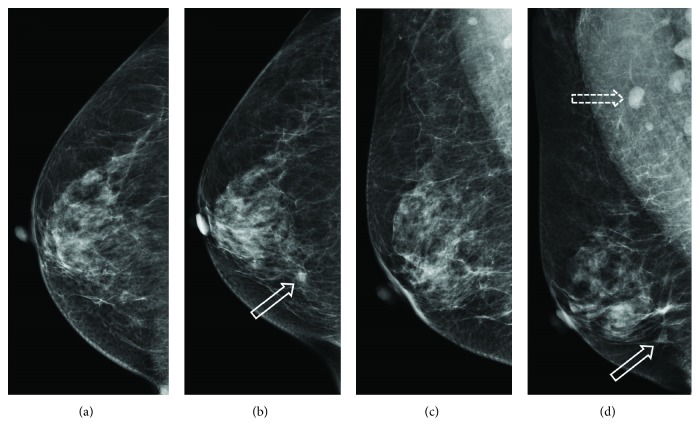
Mammography. (a and c) No suspicious lesions were detectable on June 25, 2012. (b and d) An about 7 to 8 mm focal asymmetry (solid arrows) at the posterior inner hemisphere of right breast and several enlarged lymph nodes at right axillary region (dashed arrow), diameter up to 12 mm, were noted on September 14, 2017.

**Figure 8:**
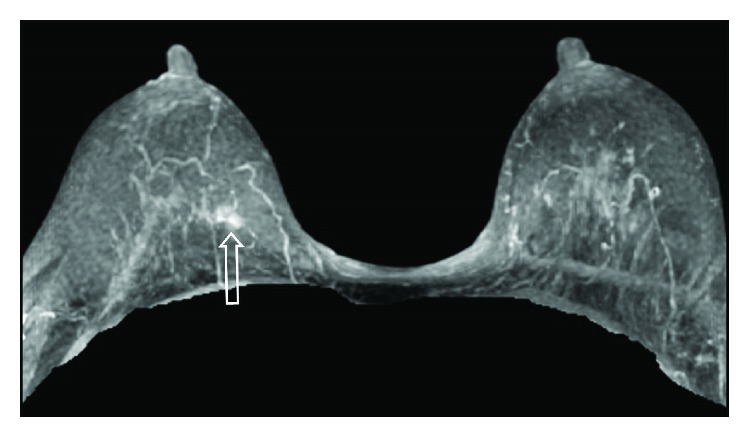
3D maximum intensity projection (MIP) contrast subtraction study MRI of breast on September 15, 2017. There was an about 7 mm focal asymmetry at the posterior inner hemisphere of right breast (arrow).

Mammogram brought to light a suspect primary site in the right breast as compared with films taken five years earlier for cancer screening (Figure 7). The lesion was also detectable in magnetic resonance imaging (MRI) (Figure 8). Nonetheless, no tumor was found by both physical examination and sonogram of breasts. Additionally, the patient's husband and sons did not want her to know the nature of the disease, so a breast biopsy was pitifully not done at last.

**Figure 9:**
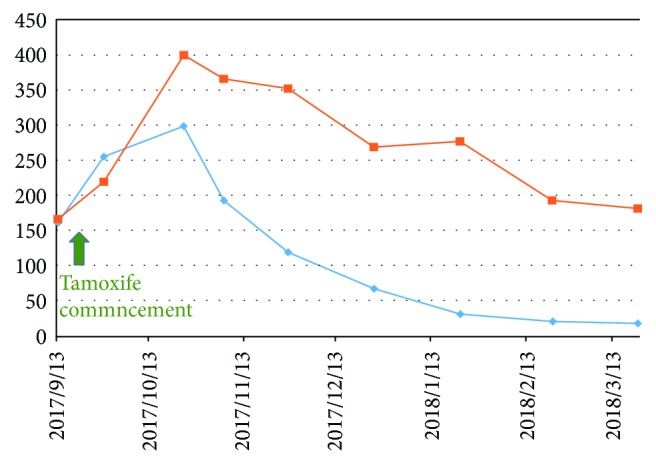
Change of serum CA 15-3 (blue diamond) and alkaline phosphatase (orange square) levels (iu/ml) along the clinical course. Green arrow: tamoxifen commencement date (September 21, 2017).

**Figure 10:**
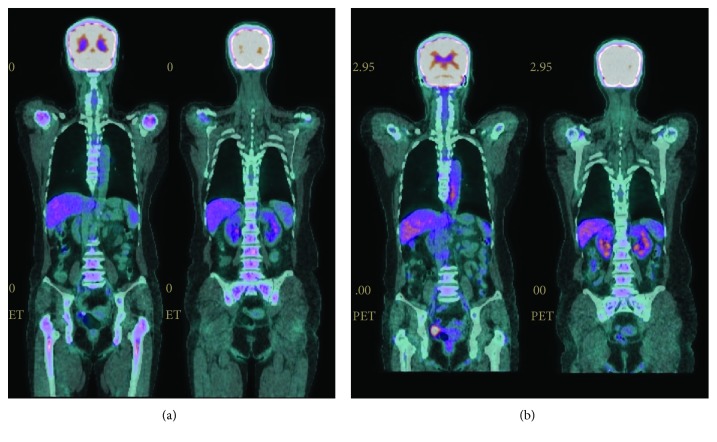
PET/CT scans. (a) Profuse bone marrow uptake (SUVmax: liver 3.1, skeleton 4.2) 15 days after starting treatment with tamoxifen, October 16, 2017. (b) Obvious resolution of previous bone marrow uptake with some residual activity in the lumbar spine (SUVmax: liver 3.3, skeleton 3.2) while on continuous tamoxifen treatment, March 5, 2018.

Due to our worry about the risk of fracture associated with aromatase inhibitors [8], the patient began to take tamoxifen, 20 mg twice daily, for treatment. The effect seemed very satisfactory. After a period of probable “tamoxifen flare” with elevation of serum CA 15-3 and alkaline phosphatase levels for six weeks [9], both of them dropped gradually within four months (Figure 9). Fortunately, there was no hypercalcemia noted during the whole clinical course. Serum CA15-3 was within normal limits at the last follow-up on March 21, 2018. Positron emission tomography/CT (PET/CT) scans during the treatment course confirmed an impressive improvement of the bone marrow uptake. The mild activity remained in the lumbar spine could be either residual metastasis or, more favored by us, compensatory hyperactive hematopoiesis in its recovery phase (Figure 10). The patient did not have headache or fever anymore and was enjoying a happy life up to the time of submitting this case report with a hemoglobin level of 12.2 g/dl.

## 3. Discussion

Occult micrometastasis to bone marrow in early stage breast cancer appeared in around 30% of the patients and was considered a poor prognostic factor [10]. Symptomatic diffuse bone marrow metastasis, on the contrary, is a relatively uncommon manifestation of breast cancer, estimated to happen in only 0.17% of the patients [5]. Most diffuse bone marrow metastasis took place in patients already diagnosed to have breast cancer. The median time from initial diagnosis of breast cancer to bone marrow involvement was reported to be from 36 to 46 months with anemia as the most frequent symptom at presentation [5, 11]. Our patient's presenting symptoms also included anemia, compatible with aforementioned reports, but her breast cancer remained almost as an unknown primary site. This scenario is indeed quite rare.

Despite that a bone marrow metastasis usually leads to an incurable stage of malignant disease, adequate treatment with endocrine agents, chemotherapy, and targeted therapy might still prolong survival in case the origin is the breast [12]. Our patient turned out to have a very successful treatment result with tamoxifen, which could be predicted from the absence of HER2 and the strong ER/PR expression in her tumor cells [13]. In the long run, when the disease becomes resistant to the present tamoxifen therapy, or even a prominent primary site lesion develops, a rebiopsy of metastatic lesions and total excision of the primary tumor are planned for investigation of possible characteristic changes of the tumor. At that time, if the cancer is still hormone receptor positive, aromatase inhibitors, selective estrogen receptor degradators, mechanistic target of rapamycin (mTOR) inhibitors, and cyclin-dependent kinase 4/6 inhibitors would accordingly be prescribed in sequence or in combination to benefit this postmenopausal patient most in regard of her quality of life [14].

Bone window of the patient's CT scan disclosed not only bone marrow involvement but also bony destruction by her metastatic cancer. It is believed that receptor activator of nuclear factor kappa-B (RANK) and its ligand (RANKL) play an important role in activating osteoclasts which constitute a vicious cycle with tumor cells to make a favorable bone marrow microenvironment facilitating bony destruction and tumor growth. Furthermore, recent studies pointed out that RANKL participates in progesterone-mediated breast cancer development [15]. Anti-RANKL monoclonal antibody, denosumab, which was more efficient than zoledronic acid in preventing skeletal events in patients with bony metastasis from breast cancer and might have direct antitumor effect against breast cancer [16], thus should be integrated into the treatment plan for patients like ours if it is reimbursed in the medical insurance system.

Cancer metastasis develops through a multistep process involving complex cell-cell interactions in microenvironments of both original and metastatic sites. Seeding into bone marrow and resulting in bony metastasis need not only RANK-RANKL pathway but also many other biologic targeting and stimulating molecules to establish an ample bone-specific tropism, fuel osteolysis, osteoblastogenesis, and T-cell differentiation [17]. Candidate genes promoting bone-specific metastasis from breast cancer have been deeply investigated. One of them was chemokine interleukin-1*β* (IL-1*β*) which drives breast cancer cell growth, colonization in the bone marrow adipose tissue compartment, and bony metastasis [18, 19]. Whether our patient's tumor produces more IL-1*β* than her normal breast tissue is currently unknown, but the possibility of treating bone marrow metastasis from breast cancer with interleukin-1 receptor antagonist (IL-1Ra), for example, anakinra, cannot be overlooked [20]. Estradiol increases IL-1*β* and decreased IL-1Ra in normal breast tissue. On the contrary, tamoxifen increases IL-1Ra significantly [21] and might to some degree act through this mechanism to achieve its amazing treatment effect in this patient.

Mucin 1 (MUC1) is a transmembrane glycoprotein functioning as an adhesion molecule which, in its circulating form, is identified as the serum tumor marker CA 15-3. MUC1 is confined to the basolateral surface of normal epithelial cells. Loss of this polarity during carcinogenesis makes MUC1 overexpressed in a hypoglycosylated form all over the surface of cancer cells, closely associated with growth factor receptors [22]. Adhesion of MUC1 to E-selectin and intercellular adhesion molecule-1 (ICAM-1) on the surface of endothelium activates Src oncogene and thus facilitates motility and metastatic potential of breast cancer cells [23]. We can assume that MUC1 is overexpressed in our patient's tumor cells based on her highly elevated serum CA 15-3 levels. Various MUC1 targeted therapies have been under development as inhibitors of cancer metastasis [24]. Despite the absence of benefit upon adding an anti-MUC1 antibody to letrozole for hormone receptor-positive metastatic breast cancer when compared with letrozole alone in a phase II trial [25], we do hope other therapeutic modalities against MUC1 might turn out to be helpful for patients like ours in the future.
